# Masculinization of the X Chromosome in the Pea Aphid

**DOI:** 10.1371/journal.pgen.1003690

**Published:** 2013-08-08

**Authors:** Julie Jaquiéry, Claude Rispe, Denis Roze, Fabrice Legeai, Gaël Le Trionnaire, Solenn Stoeckel, Lucie Mieuzet, Corinne Da Silva, Julie Poulain, Nathalie Prunier-Leterme, Béatrice Ségurens, Denis Tagu, Jean-Christophe Simon

**Affiliations:** 1 INRA, UMR 1349, Institute of Genetics, Environment and Plant Protection, Domaine de la Motte, Le Rheu, France; 2 CNRS, UMR 7144, Station Biologique de Roscoff, Roscoff, France; 3 UPMC Université de Paris 6, Roscoff, France; 4 IRISA/INRIA Centre Rennes - Bretagne Atlantique, GenScale, Campus de Beaulieu, Rennes, France; 5 Commissariat à l'Energie Atomique (CEA), Institut de Génomique (IG), Genoscope, Evry, France; University of California Davis, United States of America

## Abstract

Evolutionary theory predicts that sexually antagonistic mutations accumulate differentially on the X chromosome and autosomes in species with an XY sex-determination system, with effects (masculinization or feminization of the X) depending on the dominance of mutations. Organisms with alternative modes of inheritance of sex chromosomes offer interesting opportunities for studying sexual conflicts and their resolution, because expectations for the preferred genomic location of sexually antagonistic alleles may differ from standard systems. Aphids display an XX/X0 system and combine an unusual inheritance of the X chromosome with the alternation of sexual and asexual reproduction. In this study, we first investigated theoretically the accumulation of sexually antagonistic mutations on the aphid X chromosome. Our results show that i) the X is always more favourable to the spread of male-beneficial alleles than autosomes, and should thus be enriched in sexually antagonistic alleles beneficial for males, ii) sexually antagonistic mutations beneficial for asexual females accumulate preferentially on autosomes, iii) in contrast to predictions for standard systems, these qualitative results are not affected by the dominance of mutations. Under the assumption that sex-biased gene expression evolves to solve conflicts raised by the spread of sexually antagonistic alleles, one expects that male-biased genes should be enriched on the X while asexual female-biased genes should be enriched on autosomes. Using gene expression data (RNA-Seq) in males, sexual females and asexual females of the pea aphid, we confirm these theoretical predictions. Although other mechanisms than the resolution of sexual antagonism may lead to sex-biased gene expression, we argue that they could hardly explain the observed difference between X and autosomes. On top of reporting a strong masculinization of the aphid X chromosome, our study highlights the relevance of organisms displaying an alternative mode of sex chromosome inheritance to understanding the forces shaping chromosome evolution.

## Author Summary

Males and females differ in their optimal values for most phenotypic traits, which makes intra-locus genetic conflicts among sexes common. Sex chromosomes have a sex-biased transmission, a pattern which might create favourable conditions for the spread of sexually antagonistic alleles (i.e. alleles beneficial for one sex but deleterious for the other). Yet, expectations for genetic systems with unusual inheritance of sex chromosomes may differ from those derived from standard systems (e.g. XY). Here we demonstrate theoretically that in organisms such as aphids, which alternate sexual and asexual reproduction and display an unusual inheritance of the X chromosome, male-beneficial sexually antagonistic alleles accumulate preferentially on that chromosome, while asexual female-beneficial alleles accumulate on autosomes. Theoretical models suggest that the evolution of sex-biased gene expression may solve such sexual conflicts, by restricting the product of a sexually antagonistic allele to the sex it benefits. We show that in the pea aphid, the genomic location (X *versus* autosomes) of genes with a sex-biased expression fits predictions derived from this hypothesis. On top of reporting a strong masculinization of the aphid X chromosome, our study highlights the relevance of organisms with an alternative mode of sex chromosome inheritance to understanding the evolutionary forces shaping chromosome evolution.

## Introduction

As males and females differ in their optimal values for most phenotypic traits, selection often runs in opposite directions in the two sexes, a situation called sexual antagonism [1]. Since males and females share most of their genome, intra-locus conflicts appear when the same gene is selected for different optima in each sex. Because sex chromosomes have a sex-biased transmission pattern they are expected to accumulate different types of sexually antagonistic mutations than autosomes, as originally shown by Rice [2] and elaborated by further models [3]–[6]. The Y chromosome is expected to accumulate alleles that are good for males (even if detrimental for females) because the beneficial effects are always achieved but not the costs. It has been shown that the Y is indeed enriched in genes with male-specific functions ([7]–[9], see [10] for W in ZW systems). The situation of the X chromosome for XX/XY or XX/X0 systems (or Z in ZZ/ZW systems) is however more complex. X-linked recessive mutations are always exposed to selection in the heterogametic sex (i.e. XY males or ZW females), and their spread in the population is thus facilitated if beneficial or impeded if deleterious for the heterogametic sex. Conversely, for a dominant or partly dominant sex-linked mutation, the homogametic sex (i.e. XX females or ZZ males) has the highest influence on the evolutionary fate of mutations, because such a mutation experiences 2/3 of the time selective pressures acting on the homogametic sex. Overall, Rice's model underlines the crucial effect of the recessive or dominant character of mutations, with the X accumulating either male- or female-beneficial mutations depending on their dominance status. Although Rice's model assumes that the dominance coefficient of each allele is the same in both sexes, which may not necessarily be the case (e.g. [3], [11]), more general models show that its predictions regarding the spread of sexually antagonistic alleles on the X *versus* autosomes still hold when dominance differs among the sexes, the outcome depending on dominance in the heterogametic sex [3]. For example in a XY species, male-beneficial alleles invade more easily the X chromosome when they are recessive in males, and the autosomes when they are dominant in males, while female-beneficial alleles invade more easily the X when they are dominant in males (and the autosomes when they are recessive in males). Interestingly, some of Rice's predictions have been experimentally validated by engineering a novel sexually antagonistic allele in *Drosophila melanogaster* using genetic constructs [12].

The evolution of sex-biased gene expression has been proposed as a possible way of resolving conflicts raised by the spread of sexually antagonistic alleles over protein-coding sequence [2], see also Box 5 in [13] (throughout, male-biased [or female-biased] genes refer to genes overexpressed in males [or females]). Indeed, once a sexually antagonistic allele is present (either segregating or fixed) in a population, any modifier of expression (not necessarily physically linked to the target) that reduces the expression of the gene in the harmed sex will be selected for and fixed, allowing the allele that favors the other sex to reach fixation too (if it is not already fixed) [2]. Although this hypothesis is frequently presented as plausible (e.g. [13]–[15]), we lack empirical demonstration that sex-biased gene expression might have been selected because it allowed to solve past sexual antagonism, presumably because of the difficulties to demonstrate that a given substitution in a genome corresponds to the fixation of a sexually antagonistic allele. Accordingly, empirical support for this hypothesis is at best correlative: if intra-locus sexual conflicts are frequently resolved by the evolution of a sex-biased gene expression, we expect an accumulation of either male- or female-biased genes on the X (or Z), depending on the average level of dominance of sexually antagonistic mutations. This could account for the non-random distribution of genes with sex-biased expression between the X (or Z) and the autosomes observed in different groups of animals. Male-biased genes are overrepresented on the X chromosome in mammals [16]–[20], but under-represented in nematodes [21], flies [22]–[24] but see [25], mosquito [26], [27] but see [28] and flour beetle [29]. Female-biased genes are also overrepresented on the X in some species [16], [22], [29] but are under-represented in nematodes [21]. In systems where female is the heterogametic sex (i.e. ZW systems), the Z is enriched with testis genes in the silkworm [30] and birds [19], [31]–[33], but depleted from female-biased genes in birds [31], [33], [34] but see [35]. However, several other factors could explain the opposite trends observed in different species. In particular, the inactivation of the X during late spermatogenesis (Meiotic Sex Chromosome Inactivation, MSCI, [36], [37]) drives spermatogenesis genes out of the X in *Drosophila* and mammals [16], [20], [36], [38], [39]. Recent evidence for the absence of X dosage compensation in *Drosophila* testis [40] also explains the apparent paucity of genes expressed in the male germline on the *Drosophila* X [41]. Furthermore, the X in the whole body of male *Drosophila* is naturally hyper transcribed as a whole to equalize X∶A expression rate for dosage compensation [42], so that evolving further overexpression of X-linked genes in males may be difficult [43], [44]. Finally, the absence of a global mechanism of dosage compensation in birds (a ZW system) might also account for the overrepresentation of male-biased genes on the Z [45], [46]. As a result, the non-random distribution of sex-biased genes between autosomes and sex chromosomes does not in itself demonstrates that sex-biased gene expression evolved to solve past intra-locus sexual conflicts over protein coding sequences. The most convincing (through indirect) evidence for the resolution of intra-locus sexual conflicts *via* the evolution of sex-biased gene expression comes perhaps from the non-recombining old homomorphic ZW sex chromosomes of the emu, a ratite bird [14]. While it is widely accepted that the cessation of recombination between proto-sex chromosomes has been favored because of the accumulation of sexually antagonistic alleles in the vicinity of the sex-determining region [47]–[49], in the emu, the evolution of sex-biased gene expression may have provided an alternative solution to alleviate the segregation load due to sexually antagonistic alleles [14]. This could explain the occurrence of old and homomorphic sex chromosomes in ratite birds.

**Figure 1:**
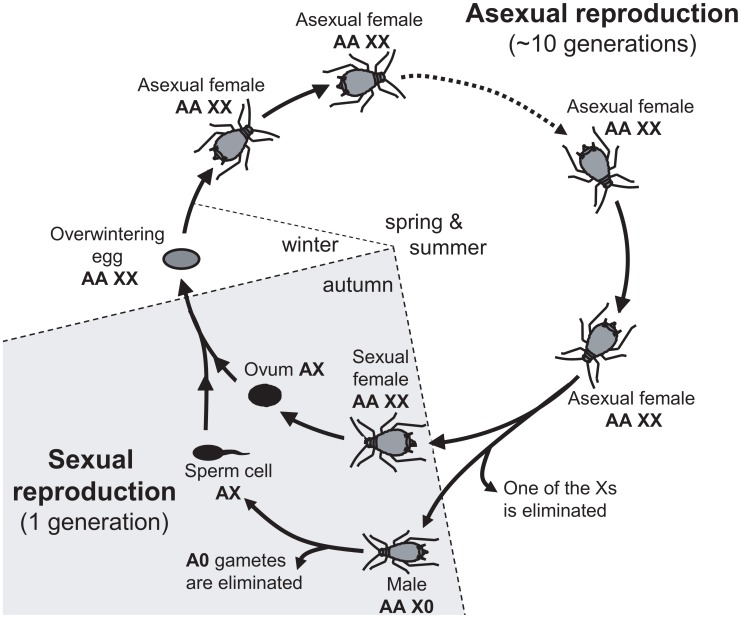
Annual life-cycle of the pea aphid and ploidy levels for autosomes (A) and sex-chromosome (X). Overwintering egg, diploid for both types of chromosomes (AA and XX) gives birth to an asexual female. After several cycles of apomictic parthenogenesis, asexual females produce sexual females and males. Males inherit the same autosomal genome as asexual females, but receive only one of the female Xs: hence they are diploid for the autosomes and haploid for the X (represented as AAX0). Ovules (haploid for both the autosomes and the X) are generated by a normal meiosis, but males produce only X-bearing sperm (AX). The fusion of male and female gametes restores the diploid level at both the X and the autosomes.

Alternative sex-determining systems are of high interest, because they allow studying the selective forces driving the different patterns from another perspective, e.g. [33], [50]. In this article, we investigate the evolutionary forces driving the chromosomal location of sexually antagonistic mutations in aphids. Aphids have an XX/X0 sex determination system whereby females carry two X chromosomes and males only one X (while both sexes are diploid for the autosomes). Yet, aphids are peculiar because in addition to males and sexual females, apomictic parthenogenetic females (diploid at the X and autosomes) represent a major component of their life cycle. This could set the stage for a three-way genetic conflict since mutations may be beneficial to either males, sexual females or asexual females. Furthermore, the alternation of asexual and sexual reproduction results in an unusual (autosome-like) inheritance of the X (see Figure 1 and [51]). During the first part of the cycle (spring and summer), asexual females (XX/AA) reproduce through parthenogenesis. In autumn, asexual females generate males and sexual females in response to photoperiodic cues: sexual females are therefore strict clones of asexual females (hence also XX/AA), while one of the X is lost to generate X0/AA males [52]. The fusion of an ovule (haploid for the X and for the autosomes) and a sperm (always haploid for the X and for the autosomes because males produce only X-bearing gametes) restores diploidy at both Xs and autosomes to generate an egg from which an asexual female will hatch in spring (Figure 1). Hence, the X is transmitted equally through males and sexual females in aphids: one half of the Xs found in the sexual progeny comes from the mother and the other half from the father (i.e. the X have an “autosome-like” inheritance, Figure 1, see also [51]). This contrasts with standard XY systems, where the X is transmitted twice more often through females than through males. These differences between aphids and standard systems have been shown to influence the neutral diversity and gene evolutionary rates of the X chromosome in aphids [51], and could also affect the evolutionary forces that promote the accumulation of sexually antagonistic mutations on sex chromosomes in standard XX/XY or ZZ/ZW systems [2], .

Here we investigate how the particular inheritance of the X, and the alternation of sexual and asexual reproduction involving specialized reproductive morphs (males, sexual females and asexual females) affect the location of sexually antagonistic mutations (the term sexually antagonistic mutation when applied to aphids refers to a mutation beneficial for at least one of the three reproductive morphs and deleterious for at least one of the two others). Using a modeling approach, we show that: 1) sexually antagonistic mutations beneficial for males – detrimental to asexual females are expected to accumulate preferentially on the aphid X chromosome, 2) mutations beneficial for asexual females – detrimental for males are expected to accumulate preferentially on autosomes, 3) the selective effect of a mutation in sexual females has little effect upon its genomic location, and 4) in contrast with previous results derived for standard systems, these qualitative predictions are unaffected by the dominant or recessive character of mutations. Under the hypothesis that the evolution of sex-biased expression to restrict the product of sexually antagonistic allele to the sex it benefits might solve intra-locus sexual conflicts, one expects that male-biased genes should be enriched on the X while asexual female-biased genes should be enriched on autosomes. Using gene expression data (RNA-Seq) in males, sexual females and asexual females of the pea aphid, we confirm these theoretical predictions.

## Results

### The X Chromosome Accumulates Sexually Antagonistic Mutations Beneficial to Males, and Autosomes Mutations Beneficial to Asexual Females

Using a general model in which a given mutation (denoted *B*) may have different effects on the fitnesses of asexual females, sexual females and males (see Table 1, Methods), one predicts that an autosomal mutation increases in frequency when rare if
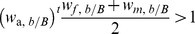
where *t* is the number of clonal generations per cycle and w_a,*b/B*_, w_f,*b/B*_ and w_m,*b/B*_ are the fitnesses of heterozygous asexual females, sexual females and males, relative to the fitnesses of individuals homozygous for the ancestral allele. When the mutation occurs on the X chromosome, this condition becomes
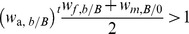
where w_m,*B/*0_ is the fitness of hemizygous males carrying the mutation. Using the notation of Table 1, we have w_m,*b/B*_ = 1+*h_m_ s_m_* and w_m,*B/*0_ = 1+*s_m_*. Assuming that 0<*h_m_*<1, the condition for invasion of a male-beneficial mutation (*s_m_*>0) is therefore more stringent when this mutation occurs on an autosome than when it occurs on the X chromosome. Conversely, the condition for invasion of a male-detrimental allele (*s_m_*<0) is more stringent when it occurs on the X chromosome than on an autosome. Among the mutations selected differentially between the sexes, one thus expects an excess of male-beneficial, female-detrimental mutations on the X chromosome, and an excess of female-beneficial, male-detrimental mutations on autosomes. Note that these expectations are not affected qualitatively by the dominance coefficients of mutations, and thus differ from those derived for standard XX/XY sex-determining systems [2], for which opposite results are found for dominant or recessive mutations. Finally, it is important to note that selection coefficients of mutations in asexual females (*s_a_* in Table 1) have a disproportionate effect on invasion criteria, due to the many asexual generations per cycle (exponent *t* in the equations above). Therefore, when *s_i_* (where *i* stands for *m* or *f*) has the same sign as *s_a_*, one expects that (in most cases) *s_i_* has little effect on whether the mutation spreads or not. For this reason, *s_m_* should generally have little effect on conditions for the spread of mutations with *s_m_*<0, *s_f_*>0 and *s_a_*<0 or with *s_m_*>0, *s_f_*<0 and *s_a_*>0 (the direction of selection is the same in males and asexual females, but different in sexual females). Although one would predict (based on the arguments above) that the first type of mutation (*s_m_*<0, *s_f_*>0, *s_a_*<0) is found more often on autosomes and the second type (*s_m_*>0, *s_f_*<0, *s_a_*>0) more often on the X, the bias should be rather small.

**Table 1:** Model of the effects on fitness (*w*) of a mutation.

Location of the mutation		Asexual females (AA XX)			Sexual females (AA XX)			Males (AA X0)		
Autosomes	Genotypes	*b/b*	*b/B*	*B/B*	*b/b*	*b/B*	*B/B*	*b/b*	*b/B*	*B/B*
	Fitness *w*	w_a,*b/b*_ = 1	w_a,*b/B*_ = 1+*h_a_ s_a_*	w_a,*B/B*_ = 1+*s_a_*	w_f,*b/b*_ = 1	w_f,*b/B*_ = 1+*h_f_ s_f_*	w_f,*B/B*_ = 1+*s_f_*	w_m,*b/b*_ = 1	w_m,*b/B*_ = 1+*h_m_ s_m_*	w_m,*B/B*_ = 1+*s_m_*
X-chromosome	Genotypes	*b/b*	*b/B*	*B/B*	*b/b*	*b/B*	*B/B*	*b/0*	*B/0*	-
	Fitness *w*	w_a,*b/b*_ = 1	w_a,*b/B*_ = 1+*h_a_ s_a_*	w_a,*B/B*_ = 1+*s_a_*	w_f,*b/b*_ = 1	w_f,*b/B*_ = 1+*h_f_ s_f_*	w_f,*B/B*_ = 1+*s_f_*	w_m,*b/0*_ = 1	w_m,*B/0*_ = 1+*s_m_*	-

*s_f_*, *s_m_* and *s_a_* respectively denote the homozygous or hemizygous effect of a mutation *B* present in sexual females, males or asexual females, while *h_f_*, *h_m_* and *h_a_* denote the dominance coefficients of *B* in these different types of individuals.

**Figure 2:**
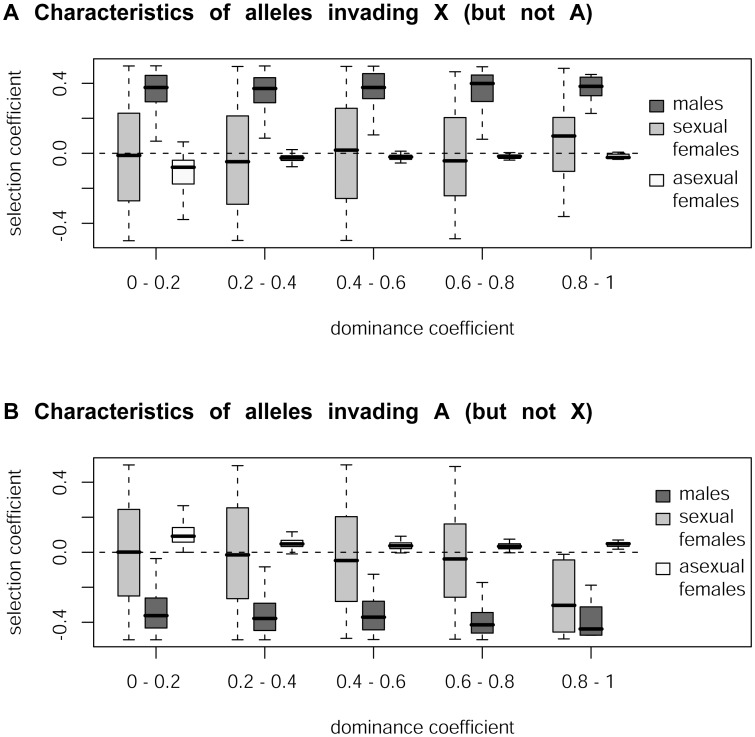
Simulation of the accumulation of sexually antagonistic mutations on X chromosome and autosomes in aphids. Characteristics of mutations (in terms of their selection coefficients in males [*s_m_*], in sexual females [*s_f_*] and in asexual females [*s_a_*]) that rise in frequency on the X more than on autosomes (panel A) or *vice-versa* (panel B) as a function of the dominance coefficient *h*. Our simulations predict that the X chromosome of aphids should be enriched in sexually antagonistic alleles beneficial for males whereas autosomes should be enriched in alleles favorable for asexual females under all dominance values.

Our simulations confirm that mutations rising in frequency on the X but not on autosomes correspond to sexually antagonistic mutations favorable for males but slightly deleterious for asexual females for all values of dominance *h* (Figure 2, see also Table S1). In contrast, mutations rising in frequency on the autosomes but not on the X are deleterious for males but slightly beneficial for asexual females. Note that when selection coefficients in asexual females are too strong, the fate of mutations becomes independent of *s_f_* and *s_m_* (again because of the larger number of asexual generations per cycle), and therefore also independent of their genomic location. When *s_f_* and *s_a_* have opposite signs, the overall effect of selection in females is attenuated (the product (w_a,*b/B*_)*^t^* w_f,*b/B*_ in the equations above becomes closer to 1), which increases the parameter range where mutations are favored in one genomic location only. This effect is visible on Figure 2 only for high values of *h*, as the overall effect of selection on rare alleles in females is enhanced when *h* is high. For lower values of *h*, selection coefficients of mutations in sexual females have little effect on their preferred genomic location. In contrast with these results on the aphid-like system, simulating a standard XX/XY sex-determining system yields the classical prediction that the type of mutation invading preferentially the X chromosome depends on whether mutations are dominant or recessive (Figure S1, see also [2]).

Additional simulations performed specifically for the aphid system showed that sexually antagonistic mutations beneficial to males – deleterious to asexual females accumulated on the X while those deleterious for males – beneficial to asexual females rose in frequency on autosomes under all tested conditions (Figure S2, Table S1). These additional results include in particular a set of simulations run under a general model of dominance (i.e. the dominance of an allele can differ between morphs, *h_a_*≠*h_m_*≠*h_f_*) and another set where beneficial (resp. deleterious) mutations are dominant (resp. recessive), as predicted by different models of stabilizing selection on quantitative traits [54].

### Expected Genomic Location of Sex-Biased Genes if Sexual Antagonism Drives the Evolution of Differential Gene Expression

Based on the previous arguments, one can deduce the preferred genomic location (X *versus* autosomes) of mutations characterized by different combinations of selective effects in males, sexual females and asexual females (Table 2, Predictions 1). We also derived the expected pattern of expression of genes bearing such kind of sexually antagonistic mutations under the hypothesis that modifiers decreasing expression in the harmed sex will be selected to solve the conflict (Table 2, Predictions 2). By combining Predictions 1 and 2, we obtained the expected chromosomal location for genes with contrasted expression patterns in the three reproductive morphs (Table 2, Predictions 3). More precisely, we expect 1) an enrichment of the X with genes overexpressed solely in males (i.e. M+F−A− genes, where M, F and A refer to male, sexual female, asexual female, respectively, and the sign represents relative expression in each morph) and with those overexpressed in both males and sexual females (M+F+A−), 2) an enrichment of autosomes with genes overexpressed in asexual females (M−F−A+) or in both asexual and sexual females (M−F+A+), 3) little chromosome bias for genes overexpressed in sexual females (M−F+A−) or in both males and asexual females (M+F−A+), with a slight autosomal bias for the former and a slight bias towards X for the latter.

**Table 2:** Theoretical predictions of the genomic location of sex-biased genes in aphids.

	Theoretical predictions:			Empirical test of Predictions 3: Genomic location of sex-biased genes				
	Predictions 1: Preferred location for mutations with antagonistic effects (based on the analytical model and on our simulations)	Predictions 2: Expression pattern of sexually antagonist mutations after the evolution of modifiers of gene expression	Predictions 3: Combination of Predictions 1 and 2	Random expectations: All genes: #X∶#A = 497∶3215, *f*_(X)_ = 0.13Genes with >5 reads: #X∶#A = 390∶2930, *f*_(X)_ = 0.12				
Fitness effect of the mutation				threshold for expression bias	#X∶#A	*f*(X)	% deviation	*X^2^* test P-value
*s_m_*>0, *s_f_*<0, *s_a_*<0	X	M+F−A−	M+F−A−: enriched on the X	2-fold	63∶205	0.24	**+100%**	**<10^−8^**
				5-fold	54∶118	0.31	**+167%**	**<10^−14^**
				10-fold	44∶87	0.34	**+186%**	**<10^−14^**
*s_m_*>0, *s_f_*>0, *s_a_*<0	X	M+F+A−	M+F+A−: enriched on the X	2-fold	6∶17	0.26	**+122%**	**0.033**
				5-fold	6∶6	0.50	**+326%**	**0.0001**
				10-fold	4∶3	0.57	**+386%**	**0.0002**
*s_m_*<0, *s_f_*>0, *s_a_*>0	A	M−F−A+	M−F−A+: depleted on the X	2-fold	11∶153	0.07	**−43%**	**0.045**
				5-fold	4∶104	0.04	**−68%**	**0.009**
				10-fold	3∶81	0.04	**−70%**	**0.020**
*s_m_*<0, *s_f_*>0, *s_a_*>0	A	M−F+A+	M−F+A+: depleted on the X	2-fold	2∶69	0.03	**−76%**	**0.019**
				5-fold	1∶21	0.05	−61%	0.29
				10-fold	1∶12	0.08	−35%	0.65
*s_m_*<0, *s_f_*>0, *s_a_*<0	Slight bias towards A	M−F+A−	M−F+A−: slight bias towards A	2-fold	19∶193	0.09	−24%	0.21
				5-fold	8∶72	0.10	−15%	0.63
				10-fold	3∶40	0.07	−41%	0.33
*s_m_*>0, *s_f_*<0, *s_a_*>0	Slight bias towards X	M+F−A+	M+F−A+: slight bias towards X	2-fold	7∶73	0.09	−26%	0.41
				5-fold	3∶51	0.06	−53%	0.16
				10-fold	2∶22	0.08	−29%	0.60

The preferred chromosomal locations of sexually antagonistic mutations (Prediction 1) are based on the simulations presented in Figure 2. Prediction 2: Predicted evolution of expression pattern of a gene bearing a sexually antagonist mutation after the evolution of a modifier that reduces the expression in the harmed sex (M, F and A refer to male, sexual female and asexual female, respectively, and the sign represents relative expression in each morph). The genomic locations of sex-biased genes (Predictions 3) were obtained by combining Predictions1 and 2. Theoretical predictions 3 were then tested with empirical data by looking at the genomic location of sex-biased genes, when considering different levels of fold-change in expression (2-, 5-, 10-fold difference). Observed number of genes for X and autosomes, frequency of X-linkage, % deviation from random expectation of X-linkage (*f*_(X)_ = 0.12) and its significance (Chi-square tests) are also given.

### Empirical Test of Predictions: The Aphid X Chromosome is Enriched in Male-Biased Genes, Autosomes Are Enriched in Asexual Female-Biased Genes

**Figure 3:**
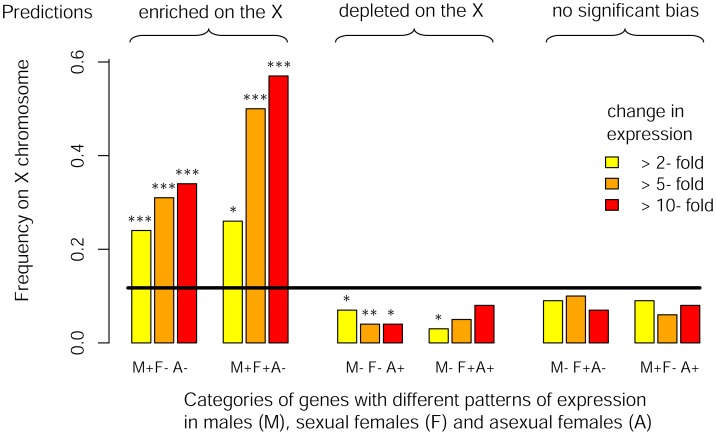
Chromosomal location of genes differentially expressed between reproductive morphs. Frequency of X-linkage for genes with different rate of expression among males, sexual females and asexual females. Genes were classified according to their pattern of expression (M, F and A stand for male, sexual female and asexual female, respectively, and the sign represents relative expression in each morph) considering different minimal fold-change in expression between reproductive morphs (2-, 5- and 10-fold). The black line shows the expected frequency of X-linkage (based on genes supported by at least 5 reads over the eight libraries). Significance for deviation from the random expectation was calculated with Chi2-tests (* : *p*<0.05, **: p<0.01, *** : *p*<0.001). Theoretical predictions for the preferred genomic location of these different classes of genes (derived under the hypothesis that the evolution of sex-biased gene expression to restrict the product of a sexually antagonistic allele to the sex it benefits might solve intra-locus sexual conflicts) are shown on the top of the figure.

We studied eight RNA-Seq libraries (three for males, two for sexual females and three for asexual females) including two previously published datasets complemented by six new libraries specifically generated for this study. We found that 5706 out of the 36990 predicted genes on the pea aphid genome were differentially expressed (*p*<0.05 after adjusting for multiple testing using the Benjamini-Hochberg method implemented in the R package DESeq) between the three reproductive morphs. When considering the 3712 genes tagged either as X-linked or autosomal (i.e. genes located within a 200 kb-window centered on the microsatellite markers tagged as X-linked or autosomal), we observed that M+F−A− genes with a 2-fold expression bias were overrepresented on the X chromosome (*f*_(X)_ = 0.24, Chi-square-test: *p*<10^−8^, Table 2, Figure 3) compared to expected proportion (*f*_(X)_ = 0.12). The bias further increased to *f*_(X)_ = 0.31 and 0.34 when considering only M+F−A− genes at least 5-fold or 10-fold overexpressed, respectively (Table 2, Figure 3). M+F+A− genes were also significantly overrepresented on the X at a 2-fold expression threshold (*f*_(X)_ = 0.26, *p* = 0.03), and at larger thresholds the effect became highly significant, the frequency of X-linkage reaching 0.50 (*p* = 0.0001) and 0.57 (*p* = 0.0002) for 5- and 10-fold overexpressed genes, respectively. By contrast, M−F−A+ and M−F+A+ genes were depleted on the X chromosome, the frequency ranging from 0.04 to 0.07 for M−F−A+ genes at different expression thresholds (*p*<0.05 in all cases). For M−F+A+ genes, the deficiency on the X was significant only at the 2-fold threshold (*f*_(X)_ = 0.03, *p* = 0.019), presumably because of lack of power due to the low number of M−F+A+ genes satisfying the 5- and 10-fold expression criteria (*n* = 22 and 13, respectively). Despite a high statistical power, genes overexpressed only in sexual females (M−F+A−) showed no significant chromosome bias at any of tested thresholds (*p* ranging from 0.21 to 0.63, *f*_(X)_ ranging from 0.07 to 0.10, Table 2, Figure 3). M+F−A+ genes also showed no significant chromosome bias (*p* ranging from 0.16 to 0.60, and *f*_(X)_ from 0.06 to 0.09, Table 2, Figure 3). Overall, the observed genomic location for genes with contrasted patterns of expression fits Predictions 3, derived under the hypothesis that the evolution of sex-biased gene expression might solve intra-locus sexual conflicts.

When similar analyses were performed considering different window sizes to assign genes to the X or the autosomes, we found fairly similar results for the 100 kb and 200 kb window. However, as the size of the window increased, the contrast between X chromosome and autosomes regarding their sex-biased gene content decreased (see Table S2). Nevertheless, the X was still significantly enriched with genes overexpressed in males (M+F−A− genes) even when no window size restriction was applied (*p* ranging from 10^−4^ to 10^−15^ depending on the fold-difference in expression considered, Table S2).

### Dosage Compensation

**Figure 4:**
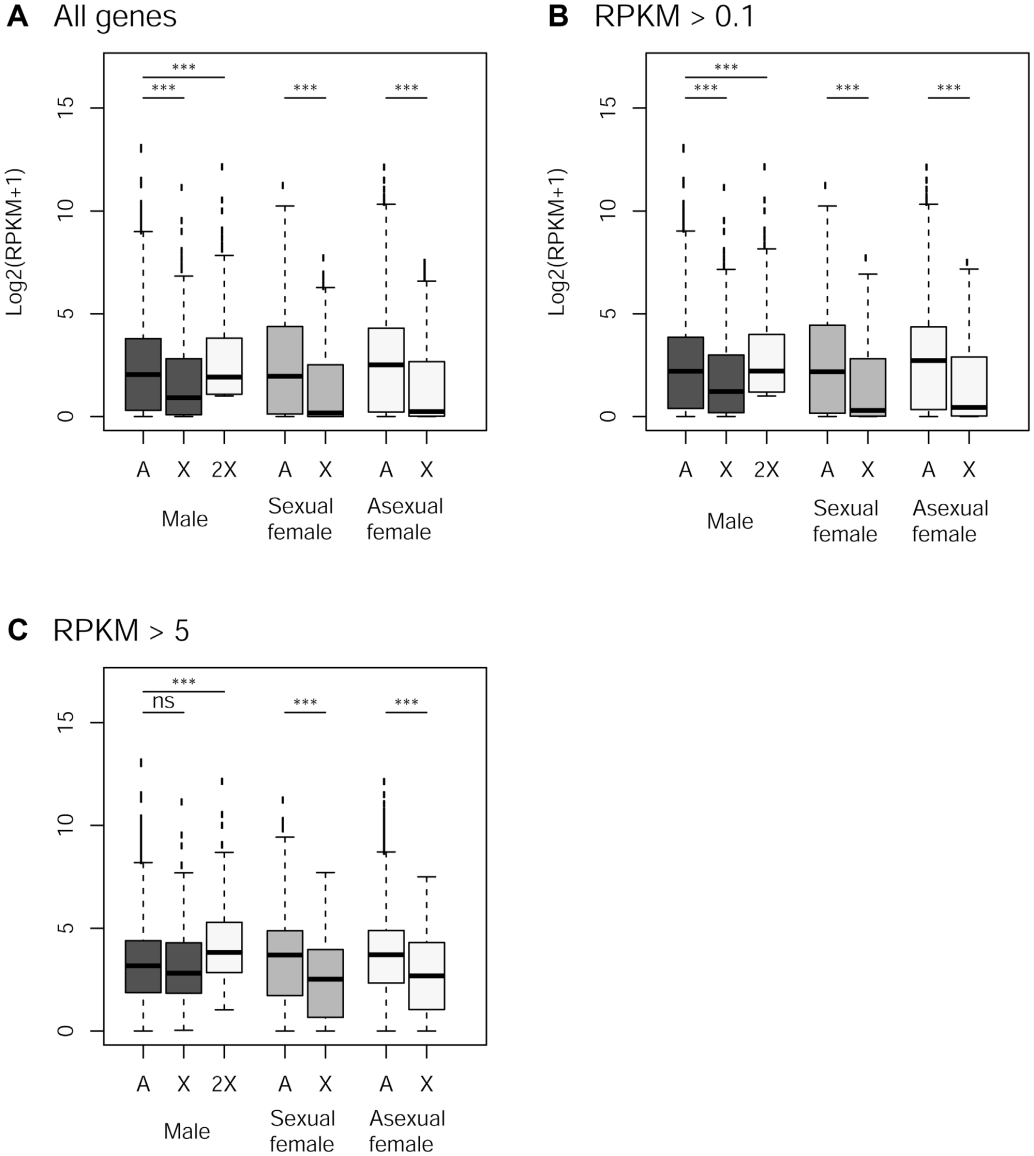
Expression rate of X-linked and autosomal genes in males, sexual females and asexual females. Panels A to C: Log2 expression (RPKM+1) for autosomal and X-linked genes in the different reproductive morphs (males, sexual females, asexual females) for different cut-offs in gene expression. The white box for males represents X-linked genes with doubled expression to account for the haploid state of X chromosome in males. Difference in gene expression between X and autosomes within each morph was tested with Wilcoxon Rank sum tests.

The frequency of low expressed genes (<0.1 RPKM, Reads Per Kilobase of exon model per Million mapped reads) on the autosomes was 5% while it reached 14% on the X (Chi-square test: *p*<10^−11^). X-linked genes were less expressed than autosomal ones in the three reproductive morphs, when considering all genes or those supported by >0.1 RPKM, Figure 4A–B, *p*<10^−7^ in all cases). In both cases, computationally doubling X-linked gene expression (to account for the haploid state of the X in males) resulted in a higher expression of X-linked genes compared to autosomal genes (*p*<10^−6^), suggesting partial dosage compensation. In contrast, X-linked and autosomal genes represented by more than 5 RPKM showed no significant difference in expression rate in males (*p* = 0.13), and computationally doubling X-linked genes expression in males resulted in significant higher gene expression for the X than for autosomes (*p*<10^−9^), suggesting a dosage compensation of these genes. X-linked genes were expressed at a much lower rate in both types of females (Figure 4C, *p*<10^−10^).

## Discussion

In this study, we demonstrate theoretically that the X chromosome of aphids is expected to accumulate sexually antagonistic alleles beneficial for males, while autosomes are expected to accumulate sexually antagonistic alleles beneficial for asexual females. We also identified a substantial masculinization of the aphid X, meaning that this chromosome is enriched with male-biased genes, and an “asexualization” of autosomes, that are enriched with asexual female-biased genes.

Our model predictions, namely the enrichment of the X with sexually antagonistic mutations beneficial for males (and the enrichment of autosomes with those favorable to asexual females) regardless of the dominance values, differ markedly from those derived for standard XY sex-determining systems, whereby the X accumulates male-beneficial female-detrimental alleles that are recessive in males (*h_m_*<0.5), and female-beneficial male-detrimental alleles that are dominant in males (*h_m_*>0.5) [2], [3], see also Figure S1. The difference between aphid and standard systems arises from the peculiar inheritance of the X in aphids (Figure 1, see also [51]) where the X is transmitted equally through males and sexual females. Male-beneficial, sexually antagonistic alleles rise in frequency on the X more easily when they are recessive in females (because their deleterious effects in asexual females are rarely expressed as long as they are rare in the population). By contrast, when *h_a_* increases, only male–beneficial mutations having minor deleterious effects in asexual females can rise in frequency on the X, since mutations that are too deleterious for asexual females will be efficiently counter-selected during the several rounds of asexual reproduction. Symmetrical arguments explain the relative enrichment of autosomes with mutations favorable for asexual females - deleterious for males (for a given *s_m_*, the effect of selection among males is weaker for autosomal loci, unless *h_m_* = 1). Differences in effective population sizes between the asexual morph (characterized by relatively small population sizes at the beginning of the asexual round of reproduction due to winter bottlenecks) and sexual morphs (characterized by large population sizes since they are generated after several rounds of clonal population growth) - that might differently affect invasion probabilities of mutations in sexual and asexual morphs through drift- are not accounted for in our analytical model. Yet, stochastic and demographic effects are incorporated in our individual-based simulations, ensuring that these predictions are robust to such effects. Aphids also contrast with other XY, X0 or ZW systems because effective population sizes for X and autosomes are similar due to their peculiar inheritance of Xs and life-cycle [51]. This implies that the fate of an X-linked or autosomal mutation should not be differentially affected by drift in aphids, contrarily to other sex-determining systems [4].

Aphids are particularly valuable systems because as shown above, the qualitative predictions for the preferred genomic location of sexually antagonistic mutations is unaffected by dominance (contrasting to standard XY systems), a parameter that is difficult to estimate for large gene sets, e.g. [55]. Furthermore, the occurrence of three different reproductive morphs in aphids (males, sexual and asexual females) creates conditions for the emergence of a three-way conflict, which allows making specific and precise predictions regarding the genomic location of sexually antagonistic genes (Table 2). Additionally, the fact that some aphid lineages have lost the ability to produce functional males and/or sexual females but still exchange genes with cyclically parthenogenetic populations [56] increases the potential for sexually antagonistic genetic variation. Indeed, deleterious alleles in sexual aphid individuals are not as strongly counter-selected (e.g. [57]) as they would be in a strictly sexual species where sexual individuals represent an obligate step for transmitting genes.

A substantial proportion of the predicted genes of the pea aphid showed differential expression between morphs (21% of the 27003 genes represented by at least 5 reads over the 8 normalized libraries were biased). Different mechanisms have been proposed to account for sexually dimorphic gene expression, including the fixation of mutations in a regulatory sequence leading to a biased expression, without the prior increases in frequency of a sexually antagonistic allele [58]–[60], epistatic interactions between sexually antagonistic alleles (which may be in protein-coding sequences [2] or in regulatory sequences [11]) and sex-limited modifiers of expression, gene duplication followed by divergence in expression of the two copies [61], [62] or genomic imprinting on allele expression dependent on its parent of origin [63]. Of course, all these mechanisms may have contributed to some extent to differential gene expression between males, sexual females and asexual females in aphids.

Our empirical analyses highlight however an important deviation from a random genomic distribution for genes differentially expressed between morphs, with male-biased genes being enriched on the X and asexual female-biased genes being enriched on autosomes.

The evolution of sex-biased gene expression may not necessarily result from the spread of sexually antagonistic alleles: a mutation that increases (or decreases) the expression of a given gene in one sex only may be favored (if it allows a better match with the optimum of that sex) even without the previous existence of an intra-locus sexual conflict. According to our theoretical predictions, a *cis-*regulatory mutation affecting gene expression in males (and thereby increasing the fitness of males) should spread more easily when it occurs on the X chromosome. However, there is no obvious reason why such male-beneficial regulatory mutations should more often increase gene expression rather than decrease it: therefore, this scenario does not explain the enrichment of male-biased genes on the X, and the enrichment of asexual female-biased genes on autosomes. Models based on gene duplication and divergence [61] should not lead to expect an excess of male-biased genes on the X in aphids, because gene duplication dampens down expression of recessive alleles in males [61], the key factor showed here to favor the spread of male-beneficial alleles on the X under a single-gene model in aphid. Sex-biased expression may also evolve by differential imprinting according to parental origin (so that male-beneficial alleles transmitted by fathers are turned off in their daughters, while female-beneficial alleles transmitted by mothers are turned off in their sons) [63], but this process is unlikely to occur in aphids given the presence of many asexual generations between each sexual event. Contrastingly, epistatic interactions between sexually antagonistic alleles over protein-coding sequence and sex-limited modifiers of expression [2] should lead to an excess of male-biased genes on the X and an excess of asexual female-biased genes on autosomes in aphids, as observed on our empirical data. Furthermore, this scenario predicts only slight chromosomal bias for sexual female-biased genes or for those underexpressed in that morph only (Table 2), and accordingly, we did not detect significant deviation from equivalent frequencies on the X and autosomes.

Our observations regarding the non-random genomic location of sex-biased genes thus suggest that sexual antagonism may have played a role in the evolution of sex-biased gene expression in aphids. Note however that we do not argue that all sex-biased genes evolved through this mechanism. We acknowledge that other models [11], [58], [61]–[66] must be invoked to explain the consequent fraction of X-linked genes (respectively autosomal genes) that are asexual female-biased (respectively, male-biased) in aphids. Hence, sex-biased gene expression does not necessarily imply a history of sexually antagonistic fitness effect. Moreover, sexual antagonism over protein-coding sequence should not systematically conduct to an evolution of sex-biased gene expression. Indeed, genes with large pleiotropic effects might be too constrained to evolve sex-biased expression [67], [68] or some genes might be too essential for allowing to cease their expression in one of the sexes, hence additional steps such as duplications would be required to solve conflicts [61], [62].

Several factors might contribute to the strong masculinization of the X in aphids, and to the differences between aphids and other invertebrate taxa. First, our models have shown that male-beneficial alleles can accumulate on the aphid X independently of dominance. Second, meiotic sex chromosome inactivation (MSCI) has been shown to drive late stage spermatogenesis genes out of the X in mammals, *Drosophila* and *Caenorhabditis elegans* [16], [36], [38], [69]. One of the several hypotheses to explain MSCI is that it evolved to prevent the spread of sex-ratio distorters on sex chromosomes [50], [70]. Since male aphids produce only X-bearing gametes (i.e. haploid for the X and for autosomes, hence the progeny from sexual reproduction is 100% asexual female), there would be no reason for MSCI to evolve or to be maintained (if this hypothesis explains MSCI). Finally, the X of *Drosophila* is not dosage-compensated in the male germline, further contributing to an apparent demasculinization of the X in this genus when relying on a 2-fold change in expression to identify male-biased genes [40], [41]. Whether dosage compensation occurs or not in the aphid male germline is unknown, but if so, this would further increase the contrast between *Drosophila* and aphids.

We examined whether our data support dosage compensation in the pea aphid whole body. We found an equal expression of X-linked and autosomal genes in males only for genes expressed at a relatively high expression (RPKM>5, Figure 4C) which would be compatible with partial dosage compensation. This could be a relic of dosage compensation that would have evolved in ancestral reproductive system in which only males and sexual females were present (i.e. before the acquisition of parthenogenesis) – partial dosage compensation has indeed been found in most XY or X0 organisms studied so far [71]–[75]. However, a surprising pattern of expression was found for females, which have equal numbers of copies of Xs and autosomes, yet showing reduced expression levels for X-linked genes, for all categories of expression level (Figure 4). Such an observation does not fit with a scenario of dosage compensation whereby differences in expression among chromosomes types are expected to be deleterious, but is best explained by a scenario involving sexual antagonism: because the phenotype of both kinds of females is probably relatively close compared to males, it is likely that a consequent fraction of sexually antagonistic mutations would have similar fitness effects on sexual and asexual females. Should such an antagonism be solved by the evolution of sex-specific expression biases, this could explain the under-expression of X-linked genes (compared to autosomal genes) in both sexual and asexual females (Figure 4). Nevertheless, we strongly caution that our preliminary conclusions on dosage compensation - drawn from whole body (i.e. including ovary and testis) – need to be validated by transcriptomic data from tissues unaffected by sex-specific evolutionary forces.

Here, by modeling the preferred genomic location of sexually antagonistic mutations in species characterized by: 1) an unconventional inheritance of the X chromosome and 2) the presence of different reproductive morphs (males, sexual females, asexual females) rather than just two sexes, we have been able to formulate several predictions regarding the genomic location of genes differentially expressed among morphs, under the hypothesis that the evolution of sex-biased expression to restrict the product of a sexually antagonistic allele to the sex it benefits might solve intra-locus sexual conflicts [2]. We then found a non-random genomic distribution of sex-biased genes that fits predictions derived from our model. Furthermore, we reported a strong masculinization of the X chromosome, contrasting with the general demasculinization of the X in all non-mammal species investigated so far and argue that it is likely due to its peculiar inheritance pattern. This study therefore highlights the relevance of organisms with peculiar modes of inheritance of sex chromosomes, such as aphids and some nematodes [76], [77], as complementary models to study the forces driving the evolution of sex chromosomes.

## Methods

### Theoretical Predictions for the Preferred Genomic Location of Sexually Antagonistic Mutations in Aphids

We used a one-locus, two-alleles model to track the spread of a sexually antagonistic mutation under an aphid life cycle. The first part of the life cycle consists in a number *t* of discrete, clonal generations; then, sexual females and males are generated and reproduce sexually (we assumed that mating is random). Two alleles *b* and *B* segregate at a given locus, and have different effects on the fitnesses of asexual females, sexual females and males (Table 1). We assumed that selection occurs among asexual females at each clonal generation, while it occurs among females and among males during the sexual phase. In a very large, randomly mating population, the spread of allele *B* from rarity is determined by its effects in heterozygous (or hemizygous) individuals: w_a,*b/B*_, w_f,*b/B*_, w_m,*b/B*_ (locus on an autosome) or w_m,*B/*0_ (locus on the X chromosome) - see Table 1. Assuming that the frequency *p* of allele *B* is small, the change in frequency over the full life cycle is approximately (to the first order in *p*):
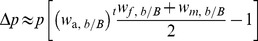
when the locus is located on an autosome, and
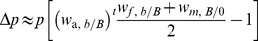
when the locus is on the X chromosome. From these expressions, predictions on the preferred genomic location of different types of mutations can be derived (see Results).

We also used individual-based simulations written in *R* [78] to explore the spread of allele *B* in a more realistic model incorporating stochasticity and demographic effects. For each replicate of the simulation, the selective coefficients *s_f_*, *s_m_* and *s_a_* (see Table 1) are randomly and independently drawn from a uniform distribution between [−0.5, 0.5]. Depending on the sampled values, allele *B* can thus be 1) beneficial for all morphs (i.e. *s_a_*, *s_f_*, *s_m_*>0), 2) deleterious for all morphs (i.e. *s_a_*, *s_f_*, *s_m_*<0), 3) sexually antagonistic if the mutation is beneficial for at least one morph and deleterious for at least one other (e.g. *s_m_*>0 but *s_a_*<0). For simplicity, we assumed that the dominance coefficient *h* of allele *B* is the same in all three morphs and is drawn from a uniform distribution between [0,1]; however we relaxed the hypothesis of identical dominance coefficients in some simulations (see below). For each combination of selection coefficients, two cases were simulated: (i) mutation *B* is carried by an autosome, (ii) mutation *B* is carried by the X chromosome. At generation 0, *N* = 1000 asexual females are created: the number of individuals of each genotype (*b/b*, *b/B* or *B/B*) is drawn from a multinomial distribution assuming Hardy-Weinberg equilibrium and an initial frequency of allele *B* of 0.005 (hence, on average 10 mutant alleles *B* are present at generation 0). Then, females reproduce through apomictic parthenogenesis for *t* = 10 generations. At each round of asexual reproduction, the number of individuals *I_i_* generated by each asexual female of genotype *i* is drawn from a Poisson distribution, with mean 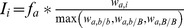, where the term *f_a_* represents the fecundity of asexual females (*f_a_* = 2) and the second term is the relative fitness of asexual female of genotype *i*. After these 10 generations, each female gives birth (by parthenogenesis) to one sexual female and one male (which carry the same diploid autosomal genome as their asexual parent). The number of gametes generated by each sexual female with genotype *i* is then sampled from a Poisson distribution with parameter 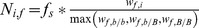, where *f_s_* is fecundity (set to 5) and the second term is the relative fitness of females with genotype *i*. If mutation *B* is located on an autosome, the number of gametes produced by each male is sampled from a Poisson distribution with parameter *N_i_*_,*m*_, which takes the same form as *N_i_*_,*f*_ (replacing *f* by *m* subscripts). If the mutation is carried by the X chromosome, *N_i_*_,*m*_ is given by 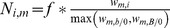. Finally, 1000 male and 1000 female gametes are randomly drawn from the pool of gametes to generate the 1000 asexual females of the next cycle. Each simulation runs for 100 cycles (a cycle including 10 rounds of asexual reproduction followed by one event of sexual reproduction), and we recorded the frequency of the mutant allele *B* in asexual females after these 100 cycles. To obtain an accurate estimate of the frequency of the mutant allele at generation 100, mutant allele frequency was averaged over 25 replicates (run with identical selection and dominance coefficients).

We tracked mutations that have opposite fates in the different types of chromosome. We considered a mutation *B* as rising in frequency on the X but not on autosomes if the frequency of the mutation at generation 100 (averaged over 25 independent runs with identical selection and dominance coefficients) increased at least ten-fold on the X (i.e. reached an average frequency of 0.05 when on the X) but was lower than 0.005 when on autosomes. Reciprocally, mutations that reached frequencies higher than 0.05 on autosomes but lower than 0.005 on the X were considered as specifically rising in frequency on the autosomes. The characteristics of such mutations (i.e. *s_a_*, *s_f_*, *s_m_*, *h*) were recorded. To explore a large panel of combinations of selection (*s_a_*, *s_f_*, *s_m_*) and dominance (*h*) coefficients, we repeated this procedure (including the simulation of 25 replicates for both types of chromosomes) by randomly drawing 200,000 sets of *s_f_*, *s_m_*, *s_a_* and *h* values.

To contrast expectations for aphids with those for standard XX/XY or ZZ/ZW sex-determining systems [2], we simulated the evolution of a newly appeared mutation *B* in standard systems 1) on the X and 2) on autosomes. In that case, the population consisted of 500 males and 500 females. The amount of gametes produced by males and females was proportional to their relative fitness values. Then 1000 male gametes (500 A/Y and 500 A/X) and 1000 female gametes were randomly drawn to generate the 500 males and 500 females of the next generation. Mutations were defined by their selective effects in males and females (*s_m_* and *s_f_*, respectively) and by their dominance value *h*. Mutations invading X but not autosomes and *vice versa* were identified as previously.

Finally, we ran additional simulations for the aphid system (with identical settings as for the core set of simulations, except for specified parameters) to extend our range of parameters. First, we relaxed the assumption of equal dominance value in the three reproductive morphs (by allowing *h_a_*≠*h_f_*≠*h_m_*) since the dominance coefficient of a mutation might differ between sexes or morphs [3]. Second, we introduced a constraint between *h_i_* and *s_i_* (where *i* stands for *a*, *f*, *m*), so that beneficial alleles are dominant (*h_i_* = 0.75 for *s_i_*>0), and deleterious ones, recessive (*h_i_* = 0.25 for *s_i_*<0). Third, we simulated a mechanism of dosage compensation similar to mammals, by assuming a dominance coefficient of *h_a_ = h_f_* = 0.5 for X-linked mutations in sexual and asexual females to model the random inactivation of one of the Xs. Fourth, we tested the influence of similar selective effects in sexual females and asexual females (i.e. *s_a_* = *s_f_*, and *h_a_* = *h_f_* = *h_m_*) since the phenotype of these two morphs are more similar compared to males. Finally, we analyzed the effect of the asexual phase length. The annual cycle was reduced to just one generation (instead of 10) of asexual reproduction directly followed by one sexual generation.

### Genomic Location of Genes with Sex-Biased Expression

Gene expression level in the three reproductive morphs was estimated from RNA-Seq data (Illumina, Illumina RNA-Seq protocol) collected on whole body of males, asexual and sexual females from the LSR1 pea aphid reference clone. For this, aphids were reared on broad bean *Vicia faba* at low density (less than five individuals per plant) to prevent the production of winged morphs. Parthenogenesis was maintained under a 16 h photoperiod and a temperature of 18°C. Twenty asexual females were then directly frozen into liquid nitrogen and kept for subsequent RNA extractions. The production of sexual individuals was initiated by transferring larvae from a 16 h to a 12 h photoperiod at the same temperature of 18°C [79]. Two generations later, sexual females and males were observed. A total of 20 adult sexual females and 20 adult males were then directly frozen into liquid nitrogen. RNA extractions were then performed using the SV Total RNA Isolation System (Promega) according to manufacturer's instructions. For each reproductive morph, 4 separate RNA extractions of 5 adult individuals were performed, for a total of 12 RNA samples. RNA quality was checked on Bioanalyzer (Agilent) and quantified on Nanodrop (Thermo Scientific). For each morph, two samples made of a pool of 2 µg of two of the four independent RNA extractions were generated, so that six RNA samples (two samples for each morph) were subsequently sent to GATC Company for RNA paired-end sequencing. RNA sample for two additional samples of male and asexual female of the LSR1 clone previously obtained using the same protocol and sequenced at the Baylor College of Medecine, USA (available in AphidBase [80] and NCBI) were also used. We thus have a total of eight RNA-Seq libraries, corresponding to three libraries for males, three for asexual females and two for sexual females (see Table S3). Reads from each library were mapped to the V2 assembly of the pea aphid genome using GSNAP [81], after filtering for rRNA. Then we recorded the number of reads as a proxy for gene expression levels in the three reproductive morphs for all 36,990 predicted genes (gene predictions 2.1 [82]). The numbers of mapped reads per library ranged from 12 to 22 millions (Table S3). We used the R package DESeq [83] to normalize the libraries (default parameters) and to identify genes showing significant biased expression between the three morphs, considering the different libraries for each morph as replicates. Significance for biased expression between reproductive morphs for each gene was calculated in DESeq. This was done by comparing two Generalized Linear models (GLM), considering or not an effect of the *reproductive morph* factor on expression level of the gene (this factor having three levels: male, sexual female, asexual female). If the inclusion of *reproductive morph* improved the model fit for a focal gene, we concluded that the morph significantly affected expression. Genes differentially expressed (*p*<0.05 after adjusting for multiple testing using the Benjamini-Hochberg method implemented in the R package DESeq) were then classified according to their pattern of expression: M+F−A− (respectively M−F+A− and M−F−A+) stands for genes at least *n*-fold overexpressed in males [M] (respectively asexual females [A] and sexual females [F]) compared to each of the two other morphs. M−F+A+ (respectively M+F−A+ and M+F+A−) stands for genes at least *n*-fold under expressed in males (respectively sexual females and asexual females) compared to each of the two other morphs and with less than 2-fold difference in the two morphs in which it is overexpressed. This classification was performed for different threshold *n* of fold-change in expression (with *n* = 2, 5 and 10). Among genes showing a non-significant bias in expression between the different reproductive morphs, we differentiated between those supported by very few reads (<5 reads in total over the eight normalized libraries) from those expressed at higher rates. Note that we worked on normalized expression data (but not on expression *per chromosome copy*).

We then restricted the following analyses to the subset of genes assigned to the autosomes or X-linked, following the approach described in [51]. Briefly, the primer sequences of 396 microsatellite loci previously assigned to the X (52 loci) or to autosomes (344 loci) [51], plus six new X-linked loci identified from a linkage analysis in a pedigree of 250 individuals from 5 families (Table S4) were mapped to the V2 genome assembly (∼24,000 scaffolds) of the pea aphid (available on AphidBase [80]). This allowed assigning 37 scaffolds to the X and 247 to the autosomes. Eleven additional scaffolds contained at least one microsatellite locus identified as X-linked and one located on the autosomes, indicating errors in the genome assembly (this was observed in large scaffolds). The average distance between the closest X-linked and autosomal microsatellite loci assigned to the same scaffold was 543 kb (min: 183 kb, max: 1900 kb). Since the probability of assembly errors increases with the size of the scaffolds, we collected only the predicted genes located in a window of 200 kb centered on each of the 402 microsatellite loci. By doing so, we obtained a tentative collection of 497 X-linked and 3215 autosomal genes. Only 14.4% of the microsatellite markers mapped to chromosomes were X-linked (though the X represents ∼1/3 of the genome size [2n = 8, 84]), and a similar proportion of genes were X-linked (13.4%).

Non-random chromosome association (X *versus* autosomes) for genes with biased expression patterns (i.e. M+F−A−, M+F+A−, M−F−A+, M−F+A+, M−F+A− or M+F−A+) was tested with Chi-square tests by comparing observed counts of X-linked and autosomal genes for each category of gene to the proportion expected under random association. This proportion was computed as the frequency of X-linkage for genes supported by at least 5 reads (rather than to the percentage of X-linkage for the 3712 genes assigned to chromosomes) because the X is slightly enriched with genes with low RNA-Seq support (see Results).

Finally, we conducted similar analyses on the genes located within a smaller window (100 kb window) around the 402 markers used to tag regions of scaffolds as X-linked or autosomal, but also at larger windows (400 kb, 800 kb, no limitation of the size of the window, i.e. the whole scaffold is used) to test whether our conclusions remained unaffected by window size. All genes ambiguously tagged as X-linked and autosomal (because located on one of the 11 chimerical scaffolds and close to two microsatellite markers tagged to different types of chromosome) were removed from the analyses. These analyses performed with sets of genes collected at different window sizes around the X *vs* autosomal tagged markers revealed that the contrast between the X chromosome and autosomes in their sex-biased gene content decreased with increasing window size (See Results, Table S2). These results, in addition to the direct evidence that 11 scaffold are chimerical between the X and autosomes, argue for the occurrence of some errors in the V2 genome assembly for large scaffolds. Indeed, such errors would lead to an increased proportion of incorrectly assigned genes to the X and to the autosomes at larger window sizes, hence to a decrease in the contrast between the X and autosomes. While this highlights the need to improve the assembly of the pea aphid genome, this does not affect our conclusions. First, the analyses presented in the Results section were performed on genes “close” to the microsatellite markers (max 100 kb), a threshold chosen to minimize error of gene assignment but allowing sufficient statistical power. Second, any error (by falsely assigning X-linked genes to autosomes and *vice versa*) should only decrease the contrast between X and autosomes, and thus be conservative regarding our conclusions.

### Dosage Compensation

To investigate for possible dosage compensation, raw expression data for each library was transformed into RPKM (Reads Per Kilobase of exon model per Million mapped reads). Expression per gene per reproductive morph was computed as the mean expression over the two or three replicate libraries for each morph, and these data were then log2+1 transformed. Non-random chromosomal distribution of genes expressed at low rate (those with <0.1 RPKM in total over the eight libraries) was tested with a Chi-square test by comparing observed counts for autosomes and X chromosome to the frequency of X-linked genes (*f*_(X)_ = 0.134). A difference in expression [log2(RPKM+1)] between X-linked and autosomal genes within each morph was tested with Wilcoxon Rank Sum tests, considering different minimal thresholds for gene expression (no restriction, RPKM>0.1, >5 in total over the eight libraries). We also computationally doubled X-linked genes expression in males (because aphid males have one X but two autosomal copies) and tested similarly if (doubled) X-linked gene expression differed from expression of autosomal genes.

## Supporting Information

Figure S1 Characteristics of mutations (in terms of their selection coefficients in males [*s_m_*] and in females [*s_f_*]) that rise in frequency on the X but not on autosomes (panel A) and autosomes but not X (panel B) as a function of the dominance coefficient *h* in standard XX/XY sex-determining systems (e.g. *Drosophila*, mammals). As predicted [2], the X chromosome is enriched with alleles beneficial for males for recessive alleles (*h*<0.5), and with alleles beneficial for females for dominant alleles (*h*>0.5). The reverse is observed for autosomes.
(TIF)

Figure S2 Characteristics of mutations (in terms of their selection coefficients in males [*s_m_*], sexual females [*s_f_*] and asexual females [*s_a_*]) that increase in frequency on the X but not on autosomes (panel A) and on autosomes but not on the X (panel B) when dominance is constant across sexes (*h_m_ = h_f_ = h_a_*) (Scenario A, see also Figure 2), when the dominance values differ between the three aphid morphs (i.e. *h_a_*≠*h_f_*≠*h_m_*, scenario B), when there is a constraint between selective and dominance effects (*h_i_* = 0.75 for *s_i_*>0 and *h_i_* = 0.75 for *s_i_*<0, where *i* stands for *a*, *f* or *m*) (scenario C), when the alleles have similar selective effects in sexual and asexual females (i.e. *s_a_* = *s_f_*, *h_a_* = *h_f_* = *h_m_*, scenario D), when the length of the asexual phase is reduced to a single generation (scenario E) and when we assume a random X chromosome inactivation in sexual and asexual females (i.e. for X-linked allele *h_a_* = *h_f_* = 0.5, scenario F).
(TIF)

Table S1 Patterns of invasion of the X chromosome and autosomes by mutations that may differentially affect fitness of males, sexual females and asexual females, derived from stochastic individual-based simulations. These results are based on a set of 200'000 simulations for each scenario (A to F) (see Methods for additional details). The selective coefficients of mutations in the different morphs (*s_m_*, *s_f_*, and *s_a_*) were drawn from a uniform distribution between −0.5 and 0.5. A mutation was considered to invade a specific chromosome if it reached a frequency >0.05 after 100 annual cycles (conversely, if its frequency was <0.005, it was considered as not invading). Mutations have been sorted according to the sign of their selective effect in one morph only (regardless of whether these mutations are good or bad to the other morphs). Here are presented the percentages of mutations that invade i) the X chromosome, ii) the autosomes, iii) the X but not the autosomes and iv) the autosomes but not the X. As an example, the first line of the table for scenario A corresponds to the ∼100'000 simulations in which the mutation was beneficial for males (i.e. *s_m_*>0) (we do not mind here of its selective effects *s_f_* and *s_a_* in the two other morphs). Among those male-beneficial mutations 53% rose in frequency on the X, 50.2% on the autosomes, 2.2% increased in frequency exclusively on the X and none of them increased in frequency exclusively on autosomes. Under all scenarios (A to F), a larger proportion of the male-beneficial alleles invades the X than autosomes. The reverse is observed for male-deleterious alleles. When alleles are sorted according to their fitness effect on asexual females (*s_a_*), we observe that a large proportion of the asexual female-beneficial alleles are likely to invade both X and autosomes, while those that are deleterious for that morph are unlikely to increase in frequency. This effect is due to the many asexual generations per annual cycle. Nevertheless under all scenarios a lower proportion of the asexual female-beneficial alleles invades the X than autosomes (opposite patterns are observed for asexual female-deleterious alleles). Selection occurring in the sexual female has little influence under most scenarios.
(DOC)

Table S2 Genomic location (X-chromosome *versus* autosomes) for genes differentially expressed in males, sexual females or asexual females when considering different sizes of window around the microsatellite markers used to tag the genomic region as X-linked or autosomal. The number of autosomal and X-linked genes (as well as X-linkage frequency) is shown when considering all predicted genes located within the window as well as when we restricted to genes supported by a total of at least five reads over the eight RNAseq libraries. Genes showing significant differences in expression between morphs (*p*<0.05 after adjusting for multiple testing using the Benjamini-Hochberg method implemented in the R package DESeq) were classified into six categories according to their specific expression patterns in the three different morphs: M+F−A− (respectively M−F+A− and M−F−A+): genes at least *n*-fold overexpressed in males (respectively asexual females and sexual females) compared to each of the two other morphs. M−F+A+ (respectively M+F−A+ and M+F+A−): genes at least *n*-fold underexpressed in males (respectively asexual females and sexual females) compared to each of the two other morphs and with similar expression level (i.e. less than 2-fold difference) in the two morphs in which it is overexpressed. This classification was performed for different thresholds *n* of fold-change in expression (with *n* = 2, 5 and 10). For each category, we show the number of autosomal and X-linked genes, the frequency of X-linkage, the percentage of deviation from random expectation (given by X-linkage frequency for genes supported by at least five reads over the eight libraries) and its significance (Chi-square test against expected proportion). Significant deviation (*p*<0.05) shown in bold.
(DOC)

Table S3 Description of the RNA-Seq libraries used for the three different types of reproductive morphs (males, sexual females and asexual females).
(DOC)

Table S4 Primer sequences of the six additional X-linked microsatellite loci. See [51] for amplification conditions.
(DOC)
