# Supplementary figures and images for: Masculinization of the X Chromosome in the Pea Aphid
Source: PLoS Genet. 2013 Aug 8;9(8):e1003690. doi: 10.1371/journal.pgen.1003690 (PMC3738461; doi:10.1371/journal.pgen.1003690)

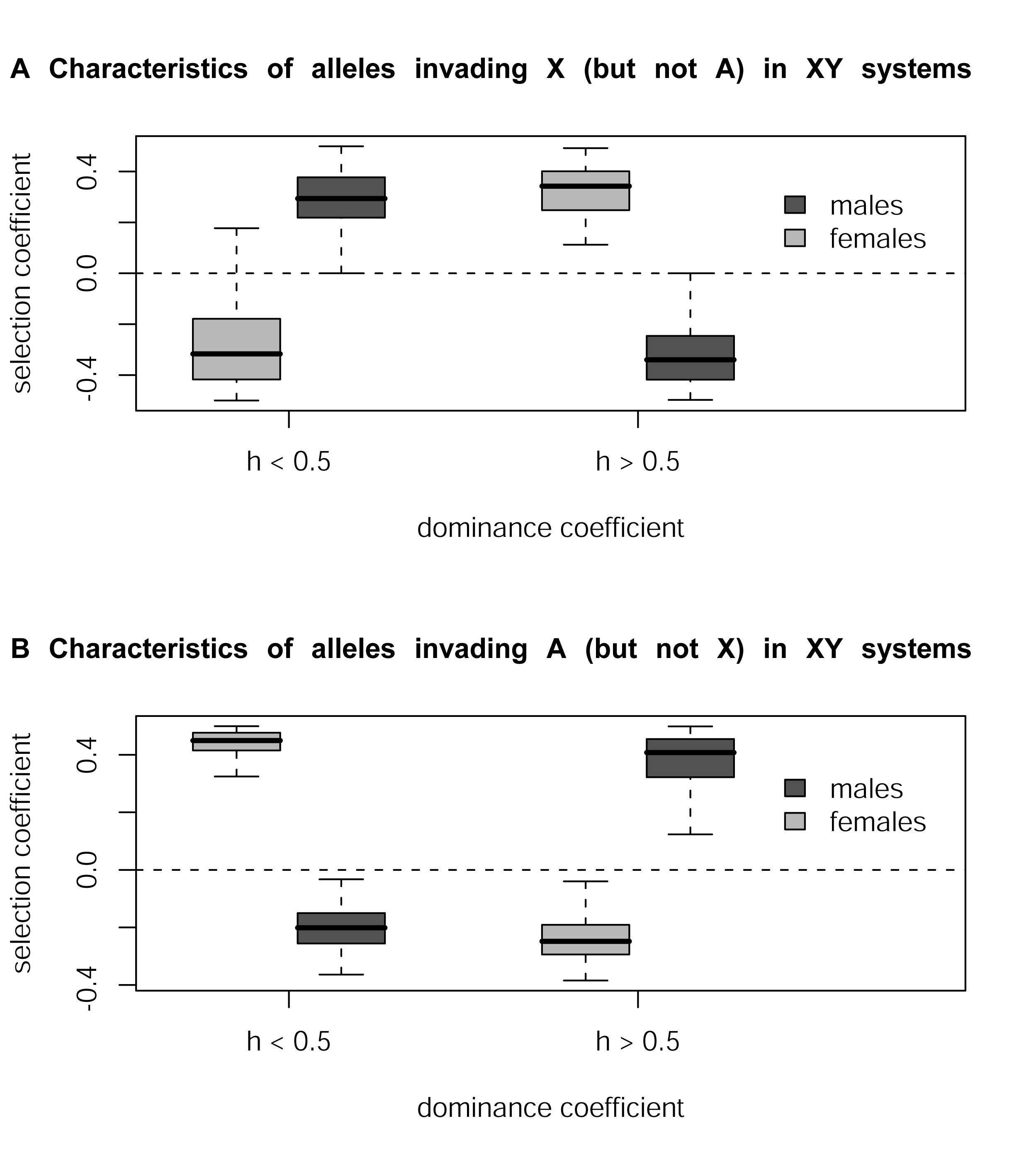

Supplement: Figure S1 — Characteristics of mutations (in terms of their selection coefficients in males [sm] and in females [sf]) that rise in frequency on the X but not on autosomes (panel A) and autosomes but not X (panel B) as a function of the dominance coefficient h in standard XX/XY sex-determining systems (e.g. Drosophila, mammals). As predicted [2], the X chromosome is enriched with alleles beneficial for males for recessive alleles (h<0.5), and with alleles beneficial for females for dominant alleles (h>0.5). The reverse is observed for autosomes. (TIF) [file pgen.1003690.s001.tif]

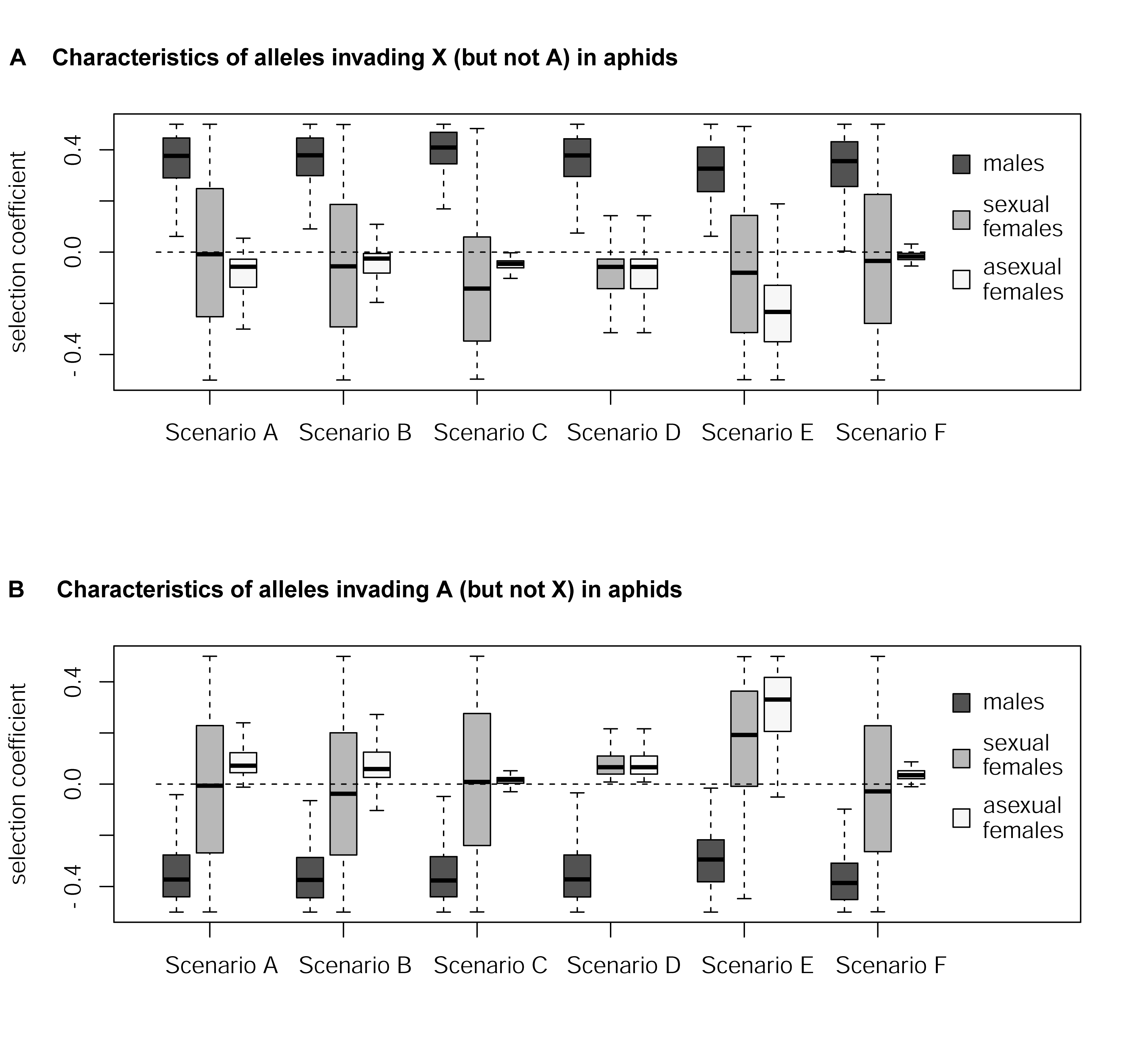

Supplement: Figure S2 — Characteristics of mutations (in terms of their selection coefficients in males [sm], sexual females [sf] and asexual females [sa]) that increase in frequency on the X but not on autosomes (panel A) and on autosomes but not on the X (panel B) when dominance is constant across sexes (hm = hf = ha) (Scenario A, see also Figure 2), when the dominance values differ between the three aphid morphs (i.e. ha≠hf≠hm, scenario B), when there is a constraint between selective and dominance effects (hi = 0.75 for si>0 and hi = 0.75 for si<0, where i stands for a, f or m) (scenario C), when the alleles have similar selective effects in sexual and asexual females (i.e. sa = sf, ha = hf = hm, scenario D), when the length of the asexual phase is reduced to a single generation (scenario E) and when we assume a random X chromosome inactivation in sexual and asexual females (i.e. for X-linked allele ha = hf = 0.5, scenario F). (TIF) [file pgen.1003690.s002.tif]
