# Supplementary material for: Masculinization of the X Chromosome in the Pea Aphid
Source: PLoS Genet. 2013 Aug 8;9(8):e1003690. doi: 10.1371/journal.pgen.1003690 (PMC3738461; doi:10.1371/journal.pgen.1003690)
Supplement: Table S1 — Patterns of invasion of the X chromosome and autosomes by mutations that may differentially affect fitness of males, sexual females and asexual females, derived from stochastic individual-based simulations. These results are based on a set of 200'000 simulations for each scenario (A to F) (see Methods for additional details). The selective coefficients of mutations in the different morphs (sm, sf, and sa) were drawn from a uniform distribution between −0.5 and 0.5. A mutation was considered to invade a specific chromosome if it reached a frequency >0.05 after 100 annual cycles (conversely, if its frequency was <0.005, it was considered as not invading). Mutations have been sorted according to the sign of their selective effect in one morph only (regardless of whether these mutations are good or bad to the other morphs). Here are presented the percentages of mutations that invade i) the X chromosome, ii) the autosomes, iii) the X but not the autosomes and iv) the autosomes but not the X. As an example, the first line of the table for scenario A corresponds to the ∼100'000 simulations in which the mutation was beneficial for males (i.e. sm>0) (we do not mind here of its selective effects sf and sa in the two other morphs). Among those male-beneficial mutations 53% rose in frequency on the X, 50.2% on the autosomes, 2.2% increased in frequency exclusively on the X and none of them increased in frequency exclusively on autosomes. Under all scenarios (A to F), a larger proportion of the male-beneficial alleles invades the X than autosomes. The reverse is observed for male-deleterious alleles. When alleles are sorted according to their fitness effect on asexual females (sa), we observe that a large proportion of the asexual female-beneficial alleles are likely to invade both X and autosomes, while those that are deleterious for that morph are unlikely to increase in frequency. This effect is due to the many asexual generations per annual cycle. Nevertheless under all scen [file pgen.1003690.s003.doc]

**Table S1**

| **Scenario A) Constant dominance across sexes (*h_m_ = h_f_ = h_a_*)** | | | | |
| --- | --- | --- | --- | --- |
|  | % of mutations that invade: | | | |
| Selection coefficient | the X | autosomes | the X but not autosomes | autosomes but not the X |
| *s_m_* > 0 | 53.0 | 50.2 | 2.2 | 0.0 |
| *s_m_* < 0 | 44.8 | 47.8 | 0.0 | 2.3 |
| *s_f_* > 0 | 50.2 | 50.3 | 1.1 | 1.2 |
| *s_f_* < 0 | 47.6 | 47.7 | 1.1 | 1.2 |
| *s_a_* > 0 | 94.4 | 97.1 | 0.1 | 2.3 |
| *s_a_* < 0 | 3.4 | 0.9 | 2.1 | 0.0 |
|  |  |  |  |  |
| **Scenario B) General model of dominance (*h_m_ ≠ h_f_ ≠ h_a_*)** | | | | |
|  | % of mutations that invade: | | | |
| Selection coefficient | the X | autosomes | the X but not autosomes | autosomes but not the X |
| *s_m_* > 0 | 52.9 | 50.8 | 1.2 | 0.0 |
| *s_m_* < 0 | 44.8 | 47.1 | 0.0 | 1.6 |
| *s_f_* > 0 | 51.0 | 51.0 | 0.5 | 0.7 |
| *s_f_* < 0 | 46.7 | 46.8 | 0.7 | 0.9 |
| *s_a_* > 0 | 94.0 | 95.7 | 0.2 | 1.6 |
| *s_a_* < 0 | 3.6 | 2.0 | 1.0 | 0.1 |
|  |  |  |  |  |
| **Scenario C) General model of dominance (*h_m_ ≠ h_f_ ≠ h_a_*) with constraints between *h_i_* and *s_i_*** | | | | |
|  | % of mutations that invade: | | | |
| Selection coefficient | the X | autosomes | the X but not autosomes | autosomes but not the X |
| *s_m_* > 0 | 53.9 | 52.8 | 0.2 | 0.0 |
| *s_m_* < 0 | 48.2 | 49.9 | 0.0 | 1.1 |
| *s_f_* > 0 | 52.5 | 52.9 | 0.1 | 0.6 |
| *s_f_* < 0 | 49.6 | 49.8 | 0.1 | 0.5 |
| *s_a_* > 0 | 97.8 | 99.0 | 0.0 | 0.9 |
| *s_a_* < 0 | 4.2 | 3.5 | 0.2 | 0.2 |
|  |  |  |  |  |
| **Scenario D) Similar selective effects in sexual and asexual females (*s_a_ = s_f_*) and constant dominance (*h_m_ = h_f_ = h_a_*)** | | | | |
|  | % of mutations that invade: | | | |
| Selection coefficient | the X | autosomes | the X but not autosomes | autosomes but not the X |
| *s_m_* > 0 | 53.4 | 50.7 | 2.1 | 0.0 |
| *s_m_* < 0 | 44.9 | 47.9 | 0.0 | 2.4 |
| *s_f_ = s_a_* > 0 | 94.9 | 97.8 | 0.0 | 2.3 |
| *s_f_ = s_a_* < 0 | 3.1 | 0.6 | 2.1 | 0.0 |
|  |  |  |  |  |
| **Scenario E) Shorter length of the asexual phase (*t* = 1 generation) and constant dominance (*h_m_ = h_f_ = h_a_*)** | | | | |
|  | % of mutations that invade: | | | |
| Selection coefficient | the X | autosomes | the X but not autosomes | autosomes but not the X |
| *s_m_* > 0 | 69.3 | 52.6 | 10.1 | 0.0 |
| *s_m_* < 0 | 17.1 | 27.6 | 0.0 | 7.1 |
| *s_f_* > 0 | 52.9 | 52.6 | 4.3 | 5.1 |
| *s_f_* < 0 | 33.9 | 27.6 | 5.9 | 1.9 |
| *s_a_* > 0 | 64.5 | 70.7 | 1.0 | 6.8 |
| *s_a_* < 0 | 22.1 | 9.1 | 9.3 | 0.2 |
|  |  |  |  |  |
| **Scenario F) Mechanism of dosage compensation similar to that found**  **in mammals (*h_a_ = h_f_ =* 0.5 for X-linked alleles)** | | | | |
|  | % of mutations that invade: | | | |
| Selection coefficient | the X | autosomes | the X but not autosomes | autosomes but not the X |
| *s_m_* > 0 | 51.5 | 50.2 | 0.7 | 0.0 |
| *s_m_* < 0 | 46.4 | 47.7 | 0.0 | 0.8 |
| *s_f_* > 0 | 50.1 | 50.1 | 0.3 | 0.4 |
| *s_f_* < 0 | 47.9 | 47.8 | 0.4 | 0.4 |
| *s_a_* > 0 | 96.2 | 97.0 | 0.1 | 0.8 |
| *s_a_* < 0 | 1.8 | 0.9 | 0.6 | 0.0 |
