# Supplementary material for: Masculinization of the X Chromosome in the Pea Aphid
Source: PLoS Genet. 2013 Aug 8;9(8):e1003690. doi: 10.1371/journal.pgen.1003690 (PMC3738461; doi:10.1371/journal.pgen.1003690)
Supplement: Table S2 — Genomic location (X-chromosome versus autosomes) for genes differentially expressed in males, sexual females or asexual females when considering different sizes of window around the microsatellite markers used to tag the genomic region as X-linked or autosomal. The number of autosomal and X-linked genes (as well as X-linkage frequency) is shown when considering all predicted genes located within the window as well as when we restricted to genes supported by a total of at least five reads over the eight RNAseq libraries. Genes showing significant differences in expression between morphs (p<0.05 after adjusting for multiple testing using the Benjamini-Hochberg method implemented in the R package DESeq) were classified into six categories according to their specific expression patterns in the three different morphs: M+F−A− (respectively M−F+A− and M−F−A+): genes at least n-fold overexpressed in males (respectively asexual females and sexual females) compared to each of the two other morphs. M−F+A+ (respectively M+F−A+ and M+F+A−): genes at least n-fold underexpressed in males (respectively asexual females and sexual females) compared to each of the two other morphs and with similar expression level (i.e. less than 2-fold difference) in the two morphs in which it is overexpressed. This classification was performed for different thresholds n of fold-change in expression (with n = 2, 5 and 10). For each category, we show the number of autosomal and X-linked genes, the frequency of X-linkage, the percentage of deviation from random expectation (given by X-linkage frequency for genes supported by at least five reads over the eight libraries) and its significance (Chi-square test against expected proportion). Significant deviation (p<0.05) shown in bold. (DOC) [file pgen.1003690.s004.doc]

**Table S2**

|  |  | 100kb | 200kb | 400kb | 800kb | No size restriction |
| --- | --- | --- | --- | --- | --- | --- |
|  | All genes | 258:1838, 0.12 | 497:3215, 0.13 | 793:5426, 0.13 | 1105:7876, 0.12 | 1308:10504, 0.11 |
| Expression pattern | Genes with >5reads | 202:1724, 0.10 | 390:2930, 0.12 | 609:4710, 0.11 | 837:6530, 0.11 | 969:8377, 0.10 |
| M+F-A-  (predicted to  enrich  on the X) | 2-fold | 34:116, 0.23  **+116%, <10^-5^** | 63:205, 0.24  **+100%, <10^-8^** | 96:375, 0.2  **+78%, <10^-8^** | 123:551, 0.18  **+61%, <10^-7^** | 125:717, 0.15  **+43%, <10^-4^** |
|  | 5-fold | 28:66, 0.30  **+184%, <10^-8^** | 54:118, 0.31 **+167%, <10^-14^** | 80:220, 0.27  **+133%, <10^-15^** | 102:341, 0.23  **+103%, <10^-13^** | 103:465, 0.18  **+75%, <10^-8^** |
|  | 10-fold | 23:45, 0.34  **+222%, <10^-9^** | 44:87, 0.34  **+186%, <10^-14^** | 65:173, 0.27  **+139%, <10^-13^** | 86:270, 0.24  **+113%, <10^-13^** | 85:369, 0.19  **+81%, <10^-8^** |
| M+F+A-  (predicted to  enrich  on the X) | 2-fold | 5:9, 0.36  **+241%, 0.002** | 6:17, 0.26  **+122%, 0.033** | 8:26, 0.24  **+106%, 0.027** | 8:36, 0.18  +60%, 0.15 | 7:53, 0.12  +13%, 0.74 |
|  | 5-fold | 5:4, 0.56  **+430%, <10^-4^** | 6:6, 0.50  **+326%, <10^-4^** | 7:10, 0.41  **+260%, <0.001** | 7:16, 0.30  **+168%, 0.004** | 7:27, 0.21  **+99%, 0.05** |
|  | 10-fold | 3:2, 0.60  **+472%, <0.001** | 4:3, 0.57  **+386%, <0.001** | 5:6, 0.45  **+297%, <0.001** | 5:9, 0.36  **+214%, 0.004** | 5:15, 0.25  **+141%, 0.032** |
| M-F-A+  (predicted to  be depleted  on the X) | 2-fold | 4:98, 0.04  **-63%, 0.030** | 11:153, 0.07  **-43%, 0.045** | 25:189, 0.12  +2%, 0.91 | 30:245, 0.11  -4%, 0.81 | 31:287, 0.10  -6%, 0.72 |
|  | 5-fold | 1:67, 0.01  **-86%, 0.015** | 4:104, 0.04  **-68%, 0.009** | 15:129, 0.10  -9%, 0.70 | 18:159, 0.10  -10%, 0.62 | 16:174, 0.08  -19%, 0.38 |
|  | 10-fold | 0:54, 0  **-100%, 0.012** | 3:81, 0.04  **-70%, 0.020** | 5:98, 0.05  **-58%, 0.036** | 7:115, 0.06  **-49%, 0.05** | 6:123, 0.05  **-55%, 0.033** |
| M-F+A+  (predicted to  be depleted  on the X) | 2-fold | 0:45, 0  **-100%, 0.022** | 2:69, 0.03  **-76%, 0.019** | 8:108, 0.07  -40%, 0.12 | 11:133, 0.08  -33%, 0.16 | 12:150, 0.07  -29%, 0.22 |
|  | 5-fold | 0:12, 0  -100%, 0.24 | 1:21, 0.05  -61%, 0.29 | 1:33, 0.03  -74%, 0.12 | 1:39, 0.03  -78%, 0.08 | 2:46, 0.04  -60%, 0.16 |
|  | 10-fold | 0:7, 0  -100%, 0.70 | 1:12, 0.08  -35%, 0.65 | 0:14, 0  -100%, 0.18 | 0:16, 0  -100%, 0.15 | 0:19, 0  -100%, 0.14 |
| M-F+A-  (predicted to show  a slight bias towards autosomes) | 2-fold | 12:113, 0.10  -8%, 0.75 | 19:193, 0.09  -24%, 0.21 | 26:277, 0.09  -25%, 0.12 | 42:363, 0.1  -9%, 0.53 | 46:445, 0.09  -10%, 0.47 |
|  | 5-fold | 6:42, 0.13  +19%, 0.65 | 8:72, 0.10  -15%, 0.63 | 11:106, 0.09  -18%, 0.49 | 19:147, 0.11  +1%, 0.97 | 19:193, 0.09  -14%, 0.50 |
|  | 10-fold | 3:24, 0.11  +6%, 0.92 | 3:40, 0.07  -41%, 0.33 | 5:62, 0.07  -35%, 0.31 | 7:89, 0.07  -36%, 0.21 | 5:124, 0.04  **-63%, 0.016** |
| M+F-A+  (predicted to show  a slight bias towards X) | 2-fold | 4:46, 0.08  -24%, 0.57 | 7:73, 0.09  -26%, 0.41 | 13:103, 0.11  -2%, 0.93 | 17:145, 0.10  -8%, 0.73 | 17:172, 0.09  -13%, 0.54 |
|  | 5-fold | 2:31, 0.06  -42%, 0.41 | 3:51, 0.06  -53%, 0.16 | 9:78, 0.10  -10%, 0.75 | 13:105, 0.11  -3%, 0.91 | 12:123, 0.09  -14%, 0.57 |
|  | 10-fold | 1:14, 0.07  -36%, 0.63 | 2:22, 0.08  -29%, 0.60 | 2:32, 0.06  -49%, 0.31 | 2:41, 0.05  -59%, 0.17 | 2:45, 0.04  -59%, 0.17 |
