# Supplementary material for: Masculinization of the X Chromosome in the Pea Aphid
Source: PLoS Genet. 2013 Aug 8;9(8):e1003690. doi: 10.1371/journal.pgen.1003690 (PMC3738461; doi:10.1371/journal.pgen.1003690)
Supplement: Table S3 — Description of the RNA-Seq libraries used for the three different types of reproductive morphs (males, sexual females and asexual females). (DOC) [file pgen.1003690.s005.doc]

**Table S3**

| Morph | Library/project ID on NCBI | # of reads mapped |
| --- | --- | --- |
| Male | SRR071347 | 19,052,145 |
|  | SRP026385 (male 1) | 22,232,030 |
|  | SRP026385 (male 2) | 19,532,914 |
| Sexual female | SRP026385 (ovipare 1) | 12,449,862 |
|  | SRP026385 (ovipare 2) | 16,405,320 |
| Asexual female | SRR073573 | 11,800,287 |
|  | SRP026385 (partheno 1) | 22,602,750 |
|  | SRP026385 (partheno 2) | 16,820,590 |
