# Supplementary material for: Masculinization of the X Chromosome in the Pea Aphid
Source: PLoS Genet. 2013 Aug 8;9(8):e1003690. doi: 10.1371/journal.pgen.1003690 (PMC3738461; doi:10.1371/journal.pgen.1003690)
Supplement: Table S4 — Primer sequences of the six additional X-linked microsatellite loci. See [51] for amplification conditions. (DOC) [file pgen.1003690.s006.doc]

**Table S4**

| **Locus ID** | **Primer F** | **Primer R** |
| --- | --- | --- |
| E_10167 | GTCGAACGCGATTTACGAAT | TCGCTTCGAACACACGTAAC |
| T_121390_2 | AATTTTGCAAATTGTTCGCC | CCGATTCGCAAAGTGGTAAT |
| E4061 | CTTTCGGAAATGCAACGTCT | TCTGACAATTAATTTTGTTTTTCATCA |
| 111910_7 | CTGTGCGCGTTTTATCTTCA | AATCGATGTCGGTCCTATGC |
| 116879_10 | CAGATTTTACACGCGTTTCG | GTATGTGCGCGTTTCGTGTA |
| E3013 | GACTCCGTCGCTTACCTGAC | TCTCATTTTTCGTCGTGCTG |
